# The Effect of Passively Viewing a Consent Campaign Video on Attitudes Toward Rape

**DOI:** 10.3389/fpsyg.2020.01741

**Published:** 2020-07-28

**Authors:** Ellie M. Rowe, Peter J. Hills

**Affiliations:** Department of Psychology, Bournemouth University, Talbot Campus, Poole, United Kingdom

**Keywords:** rape, perceptions, campaigns, attitudes, justice

## Abstract

Around 90% of rape victims know their perpetrator, making acquaintance rape the most common form of rape, contradicting societal beliefs. There is ambiguity about the meaning and use of consent in sexual scenarios (Beres, 2007). This study used a mixed methods approach to test the effectiveness of a campaign video aimed at increasing understanding of consent. We assessed whether the video affected rape judgments in vignettes depicting consensual or non-consensual sexual scenarios. We also manipulated whether making consent the primary or secondary question influenced attitudes. Text responses were also obtained to gain an insight into participant reasoning. The campaign showed no increase in rape judgments. Making consent primary in question order did lead to greater accuracy in rape judgment. A content analysis of the free-text responses indicated that the presence of the campaign actually reduced people’s use of consent in explaining why a scenario may represent rape: Instead they focused on the attractiveness of the attacker. These results are discussed in relation to the effectiveness of passively viewing campaign material.

## Introduction

Rape is typically defined as intentional penetration of the vagina, anus, or mouth of another person without their consent and when the alleged perpetrator does not reasonably believe that the person consented (Home Office, 2003). Most rape is acquaintance rape: a situation in which the rape victim knows the perpetrator (Hester and Lilley, 2016). Core to the definition of rape is consent: A person consents if the person agrees by choice and has the freedom and capacity to make that choice in United Kingdom law (Ministry of Justice Home Office Office for National Statistics, 2013), however, there are some debates in the psychological literature as to what defines consent (Beres, 2007; Muehlenhard et al., 2016). Perceptions of what consent is varies from believing that consent is any agreement to participate to others who suggest it should be freely given, but it is suggested that there is a general consensus among the population that consent is an agreement to take part in sexual activity (Beres, 2007). There are many campaigns that aim to increase public knowledge of sexual consent: This is especially important because the public comprises those who could be victims, perpetrators, bystanders, or jurors in rape trials. The effectiveness of such campaigns is limited (Brecklin and Forde, 2001). Despite this, governments, universities, and charities invest significant sums of money into developing new campaigns. It is important to assess the efficacy of such campaigns. The present study, therefore, investigates the effectiveness of a video campaign aimed at changing attitudes toward consent. It may also be important to target jurors directly because research suggests changing public attitudes with media campaigns faces many difficulties (Ogunfowokan and Fajemilehin, 2012); therefore, this study also investigates methods to increase jurors accurate judgments of whether a scenario is rape or not by establishing the importance of priming consent or rape when interpreting a sexual scenario. This determines whether an education campaign or highlighting consent is effective in increasing accurate identification of rape in sexual scenarios. Both public attitudes and the criminal justice system (CJS) are discussed in terms of their importance regarding rape occurrence and conviction.

Reporting (Temkin and Krahé, 2008) and conviction rates (Weller, 1992) for rape are drastically low. There are many potential reasons for the low reporting rate (including stigma and humiliation; Moszynski, 2004) and acknowledgment (Peterson and Muehlenhard, 2007) of rape. Many theorists indicate societal acceptance of rape myths is a strong deterrent for reporting rape and the low conviction rate (Weidner and Griffitt, 1983; George and Martínez, 2002; Sable et al., 2006). Public knowledge of rape and consent is of extreme importance due to the effects that rape can have on an individual. The effects can include but are not limited to posttraumatic stress disorder and self-blame (Peter-Hagene and Ullman, 2018), fear, anxiety, depression, and effects on self-esteem as well as sexual dysfunction (Resick, 1993). We suggest that a societal attitudinal change is necessary for a change to occur in both reporting and conviction. Reducing public acceptance of rape myths may increase reporting and conviction rates because the public is represented by jurors in the United Kingdom (Legislation gov uk, 2018) and many other democracies.

The importance of campaigns is revealed through several lines of evidence indicating that there is significant misunderstanding in what rape is by the general public, who could potentially be jurors, victims, or unintentional perpetrators of rape. Researchers have found that many aspects of a scenario can determine whether or not or how much a person perceives a scenario to be rape (Pollard, 1992; McHugh, 1996; Hills et al., 2020) such as wantedness, pleasure (Hills et al., 2020) gender, or traditionality of those who are placing the judgment (Pollard, 1992). Furthermore, respectability and attractiveness of the defendant can also influence judgment (Luginbuhl and Mullin, 1981; Tieger, 1981; Richardson and Campbell, 1982). Beres (2007) argues that society’s understanding of consent is underdeveloped and relies on assumed definitions of consent that could be challenged with appropriate campaigns. There is much ambiguity around the idea of consent, often because of the different ways in which it can be given (Hall, 1998; Humphreys, 2004, 2007) such as no response at all, verbal or non-verbal methods, and directly or indirectly (Waldby et al., 1990; Hickman and Muehlenhard, 1999; Beres et al., 2004; Humphreys, 2004). People see consent through many different behaviors and indicators in sexual scenarios that are related to wanting and pleasure, whereas others see it only as the clear verbal agreement to each sexual act (Hills et al., 2020). Others use consent as a boundary line for good and bad sex (Wertheimer, 2003). However, some suggest that the concept of consent is well understood by sexually active young adults and that people are skilled at sexual communication (Beres et al., 2004; Beres, 2007).

Many media campaigns have been developed to alter the attitudes toward rape and consent in specific groups of people. In recent years, there has been a movement from a “no means no” attitude to a “yes means yes” attitude toward consent (Beres, 2014) which is believed to be due to the increase in educational programs, activism, and media campaigns that aim to improve understanding of consent and rape (Beres, 2014). Campaigns generally aim to increase the understanding of sexual situations and, thus, consent and rape (Donat and White, 2000). Media campaigns have been effective in promoting sexual responsibility and information about consensual sex. For example Keller and Brown (2002) found that safe sex media campaigns were associated with increased teen condom use with casual partners, and other researchers have found that media campaigns have no effect on public attitude and that attitudinal changes can be hard to obtain (Brecklin and Forde, 2001; DeGue et al., 2014). Potter et al. (2009) have highlighted that poster campaigns are significantly more effective for those that have previously engaged in campaign behavior compared to those that have not. Even when campaigns have a wide reach in terms of the number of people viewing them, there is little evidence they actually change attitudes. Hills et al. (2020) have shown that passive viewing of a television campaign aimed to raise awareness of relationship abuse (“This is Abuse,” Home Office, 2003) does not increase accuracy of rating sexual scenarios as rape or not. Currently, little is known about particularly effective strategies for media campaigns (Lonsway, 1996).

If people do not fully understand consent, this may raise issues within the criminal justice system and court cases because the general public make up jurors in many Western democracies. It is important to not only try to change the opinions of the public in general who will be jurors, but also to look at ways to improve juror decision making through other means such as questioning. This is because they have the potential to contribute to the conviction rates. It is suggested that decisions made by jurors in legal cases are susceptible to social preexisting attitudes, myths, and biases toward rape and consent (McEwan, 2005). It is, therefore, important to target jurors directly to determine techniques that can influence the way jurors think before making a judgment on rape trials. Because the misunderstanding of rape is said to be surrounding consent (Beres, 2007) highlighting this element before juror decision making might have an impact on the judgment that a possible juror may make.

The present study, therefore, sees if highlighting the element of consent before a judgment is made is able to increase the accurate identification of rape in sexual scenarios. In the present study, we aim to test whether a campaign video that is widely used to highlight consent to the public (“Tea and Consent,” Blue Seat Studios, 2015), which depicts common rape myths but applied to drinking tea as an analogy to sexual intercourse, can increase accuracy in identification of rape in sexual scenarios. This video aims to highlight the importance of sexual consent in all scenarios and aims to tackle rape myths, such as assuming consent because it has been obtained previously (Monson et al., 2000) or that a lack of physical or vocal rejection is quite common in rape and sexual assault (Jozkowski, 2015). We use the scenarios devised by Hills et al. (2020) as these describe sexual scenarios that occur between two acquaintances and in which the sex can be consensual or not. In Hills et al.’s work, 65% of non-consensual scenarios were judged as representing rape, and this figure did not depend on viewing a campaign video. The current study also attempts to gain an understanding into participants’ verbal reasons for making such decisions by taking short free-text responses (a few sentences long) in addition to numerical data. The purpose of this is to provide deeper elaboration of the reasons why participants believe sexual scenarios represent rape, allowing us to identify particular themes that influence people’s judgments. We hypothesize that highlighting consent before asking participants to make a rape judgment will lead to significantly more (accurate) identification than when consent is highlighted after making a rape judgment. Further, if “Tea and Consent” (Blue Seat Studios, 2015) is successful in highlighting consent as it is intended, then participants who view the campaign video before making judgments will make significantly more (accurate) identification of rape than those who do not view the campaign video.

## Materials and Methods

### Participants

An opportunity sample of 173 participants was used, of which 90 were in the campaign-present condition and 83 were in the campaign-absent condition. Sample size was determined based on the effect size reported in Hills et al. (2020). Participants were recruited by responding to an online ad that asked potential participants if they would like to contribute to research on attitudes toward sexual violence that was distributed on survey exchange platforms. It was also advertised to students at Bournemouth University on their online research participation platform. Those from Bournemouth University were participating in return for course credits. No participant personal or demographic data was collected to ensure anonymity and to encourage participants to be completely honest in their responses. This anonymity was deliberate to protect the participants and ensure the study remained fully ethical. Participants were informed not to take part if they had any personal experience of sexual violence.

### Design

A 2 × 2 mixed subjects design was employed with two independent variables: campaign (present or absent; manipulated between subjects) and question order (consent question first or rape question first; manipulated within subjects). Question order aims to determine whether or not highlighting the concept of consent (in the form of a question asking participants to identify where it lies) before asking participants to make a judgment increases the likelihood of a rape judgment being made. The dependent variable was rape judgment of a scenario in which a response of “yes” or “no” was provided. Qualitative responses were also collected to gather an understanding into participant reasoning behind their responses (Braun and Clarke, 2013) in which participants were simply asked why they gave the answer they gave in terms of verbal reasoning. The free-text responses were approximately one to two sentences long, and an expandable answer box of approximately four lines was provided. The presentation order of the vignettes was randomized across participants such that there was a roughly equal number of consensual scenarios presented with the consent question first and the rape question first.

### Materials

Twenty-three short vignettes were used in this study developed by Hills et al. (2020). These vignettes were two to three sentences long and described hypothetical scenarios that were either consensual (11) or non-consensual (12) between acquaintances developed from the Reasons for Wanting Sex and Reasons for Not Wanting Sex questionnaire (Peterson and Muehlenhard, 2007). They were written in the second person and were gender-neutral. Hills et al. (2020) report that they had good face validity.

The campaign video used was “Tea and Consent” (Blue Seat Studios, 2015)^1^ which aimed to increase awareness of consent. This is a short video (2 min, 50 s) that demonstrates the concept of consent through the use of stick people: One of the stick people tries to force the other to drink a cup of tea in many of the scenarios that are rape myths (e.g., if they consented to drinking tea in the past, it doesn’t mean they want to now). Tea represents sexual intercourse in the video. The video was used as part of Thames Valley Police’s “Consent is everything” campaign (Thames Valley Police., 2015) which aims to raise awareness and understanding of sexual consent and suggests that, if someone is struggling to understand the idea of consent, they should imagine you’re making them a cup of tea and whether or not you’d make them drink it based on similar rape myth–type scenarios.

### Procedure

This study was granted full ethical approval from the Research Ethics Panel at Bournemouth University. The study was administered online (using Qualtrics) to encourage participants to give open, honest, anonymous responses. Participants first provided informed consent; they knew the study would involve judging sexual scenarios that may or may not be consensual. Participants in the campaign-present condition were shown the short campaign video. The survey could not move onto the next question until the video was played fully. Participants were instructed to watch the video with the sound on. This mimics passive viewing of campaigns (such as on TV) and better reflects how campaigns are viewed. Participants who were in the campaign-absent condition did not experience this.

All participants were then presented with the scenarios, one at a time in a random order. They were asked to read the scenario and to imagine themselves in the described scenario. Following each scenario, participants answered three questions concerning whether they thought the scenario displayed consent and why and whether the scenario showed rape and why. The third question was contingent on the previous responses: Participants were either asked when consent was given (if they had said the scenario was consensual) or rape occurred (if they said the scenario depicted rape). The order of the questions was manipulated between subjects with the consent question asked before the others for one group of participants. Participants answered each question on a separate page and were not allowed to access previous responses to ensure answers could not be altered. Once participants had answered the 23 vignettes, they were given debrief information, including signposting to organizations offering support.

### Data Analysis

The free-text data was analyzed using content analysis. Content analysis is a flexible method for analysis of text data (Cavanagh, 1997). The first author became familiar with the data to make sense of the data collected. Analysis was then conducted using inductive content analysis. Inductive content analysis was used because the research is not based on an earlier model (Bengtsson, 2016).

The data was first open coded, and categories were freely generated from the data. Following this, the codes were grouped into higher order codes. The aim of this was to reduce the number of similar descriptive categories in higher order categories. This allows for data to “belong” to a group that allows for comparison between groups. The process of abstraction then involved naming each group using data-congruent words that allowed for description and understanding. Once the data was in discrete categories, frequencies were collected (Bengtsson, 2016). This allowed a quantitative summary of qualitative data in addition to the depth of analysis associated with interpretation of the written text.

Triangulation was completed by the second author independently coding the data and comparing the codes. The two sets of coding were largely consistent (inter-rater reliability *r* = 0.92).

## Results and Discussion

**FIGURE 1:**
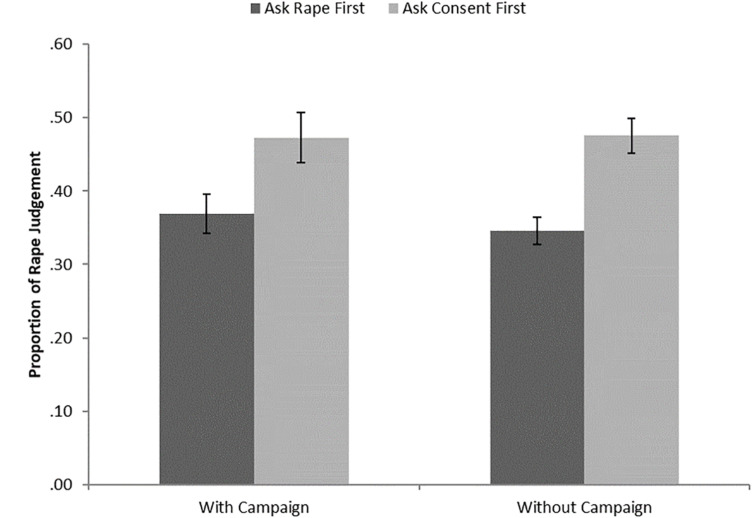
Proportion of rape judgments.

Here, we present the quantitative results, followed by the qualitative results to use these to explain the quantitative results. This form of mixed methods approach allows for a more in-depth understanding of the reasons for participant responses. Quantitative results were analyzed using a 2 × 2 mixed factorial ANOVA with the between-subjects factor of campaign and within-subjects factor of question order. The rape judgments are summarized in Figure 1.

There was a significant effect of question order on rape identification, *F*(1, 90) = 32.81, *MSE* = 0.02, *p* < 0.001, η_p_^2^ = 0.27, in which the consent-first question order led to significantly more rape judgments (*M* = 0.47, *SE* = 0.02) than the rape-first question order (*M* = 0.36, *SE* = 0.02). There was no significant effect of the campaign video on rape judgment *F*(1, 90) = 0.12, *MSE* = 0.04, *p* = 0.735, η_p_^2^ ≤ 0.01, BF_10_ < 0.01, in which the campaign-present condition had a similar number of rape judgments (*M* = 0.42, *SE* = 0.03) as the campaign-absent condition (*M* = 0.41, *SE* = 0.02). We used Bayes factor (with the prior estimated based on the significant effect of question order) to show that these results show we have strong confidence in the null hypothesis for the effect of the campaign. The interaction between question order and campaign viewing on rape judgment was not significant, *F*(1, 90) = 0.43, MSE = 0.02, *p* = 0.515, η_p_^2^ < 0.01).

**FIGURE 2:**
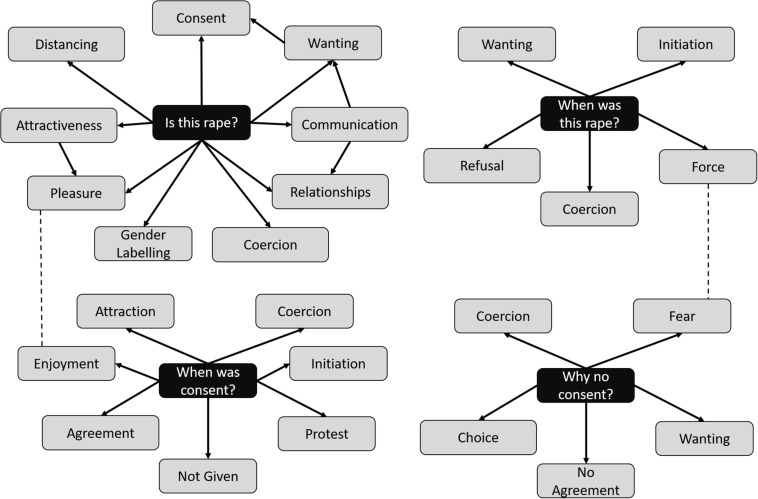
Overall picture of the resulting themes for each of the questions, highlighting some significant overlap in what determines rape and consent.

To explain these somewhat unexpected findings, we analyzed the free-text responses. We analyzed each question separately because they focused on separate issues, starting with the question about whether the participants thought the scenario was consensual or not. For the frequencies, we were able to analyze whether there was an association between the campaign presence and whether the scenario depicted consent or not. This particular analytical approach was chosen as it directly assesses the effectiveness of the campaign video. The four questions were analyzed separately as they addressed different issues. Several themes emerged from most of the questions (coercion, attraction, wanting, pleasure, and communication). Although these were subtly different across the questions, it highlights how these are core in determining whether the scenarios we presented to participants depicted consensual sex or not. Figure 2 presents an overall picture of the resulting themes for each of the questions, highlighting some significant overlap in what determines rape and consent but also some key differences. These are expanded upon here.

### Was the Scenario Rape or Not?

The category of *consent* was used more than any other category for both consensual scenarios and non-consensual scenarios (see Table 1) to describe why a non-consensual scenario may be seen as consensual. There were significantly fewer mentions of consent in the campaign-present condition for the non-consensual scenarios than in the campaign-absent condition (Table 1). This is the opposite of expected results because the video highlights the idea of consent and may suggest the video has produced a rebound effect. The rebound effect is the result of suppressing thoughts that are then likely to subsequently reappear with greater insistence than if they had never been suppressed (Macrae et al., 1994). Corrigan and Penn (1999) suggest that attempts to suppress stereotypes can lead to a rebound effect, which may explain such frequencies. Wanting was also a category that arose from the data. Participants often used consent and wanting synonymously when it came to explaining their judgments. The results from the content analysis also suggest relevance to the criminal justice system because McHugh (1996) has found that that jurors tend to ignore the absence of consent if the situation is wanted as reflected in current findings. It may be possible that an increase in understanding would help to increase conviction rates, which is important because increasing conviction may encourage higher reporting rates because many rapes go unreported due to fear of the criminal justice process and not being believed (Ryan, 2011; Wolitzky-Taylor et al., 2011).

**TABLE 1:** Summary content analysis (full analysis presented in Supplementary Table S1) for the question “Please explain why you think this scenario is rape or not rape?”

Category	Description	Mentions				Chi result
		WN	WC	WoN	WoC	
Consent	Consent was used by participants to describe why they thought a scenario was rape or not rape. For some participants, who perceived a rape scenario to be a consensual one, consent was used as their reasons, sometimes using the terms “gave in,” “eventually,” and “changing their mind” to justify the consent.	366^–^	470^+^	592	523	*X*^2^(2) = 16.43, *p* < 0.001
Wanting	Wanting was used by participants to explain why a scenario was rape or why it was consensual. This was not always in line with the presence or absence of consent. Participants sometimes used consent and wanting interchangeably as if the same thing.	43	16	48	23	*X*^2^(2) = 0.43, *p* = 0.567
Pleasure	Pleasure was used by participants to perceive sexual scenarios. Some suggested that pleasure was irrelevant to consent, and others suggested that pleasure indicated consent. Pleasure was also used alongside consent for a reason as to why a scenario was consensual.	115	87	89	45	*X*^2^(2) = 3.04, *p* = 0.088
Communication	The category of communication was used by participants to suggest why an unwanted or unpleasant scenario was not rape. Participants suggest that lack of pleasure and wanting should be discussed or vocalized before sex, in which case, this may then make the scenario rape if intercourse then occurs.	7	57	0	37	*X*^2^(2) = 4.35, *p* = 0.045
Coercion	Coercion was used by participants to suggest why a rape scenario was non-consensual. However, participants still used coercion as to why a rape scenario was consensual or why a consensual scenario was rape.	226	32	371	26	*X*^2^(2) = 6.64, *p* = 0.011
Distancing	Participants were asked to imagine themselves in the scenario and report the answers as themselves; however, when answering the questions, many participants referred to the person in the scenario in the third person, often using “you,” “they,” “the person,” or “the individual.” This appeared more for rape scenarios than consensual scenarios.	274	194	356	219	*X*^2^(2) = 1.22, *p* = 0.280
Gender Labeling	Participants were given a gender-neutral scenario, and some were also given a gender-neutral campaign and were asked to imagine themselves in the scenarios and answer it as themselves. Participants often used gender labeling in their responses with the use of “he” and “she” when referring to the scenario. It was often assumed in rape scenarios that the subject was a “he” and that the victim was a “she.”	26	21	8	7	*X*^2^(2) = 0.02, *p* = 1
Relationships	Being in a relationship was an element of the scenarios that some participants suggested was a reason as to why a non-consensual scenario was not rape. They often suggested that these things happen in relationships rather than that these scenarios are rape. Being in a relationship was suggested to be a reason as to why a rape scenario was consensual and also why a consensual scenario was consensual.	16	10	10	3	*X*^2^(2) = 0.92, *p* = 0.477
Attractiveness	Attractiveness was only mentioned twice to suggest why a consensual scenario was consensual; however, more participants suggested that being attracted to someone and finding them or their behavior appealing meant that a rape scenario was not rape.	7	2	10	0	*X*^2^(2) = 2.48, *p* = 0.211

*The table contains a description of each category and the number of mentions for each condition in the participants’ responses in their reasons for appraising situations as rape or not. Condition labels are WN, With campaign and non-consensual; WC, With campaign and consensual; WoN, Without campaign and non-consensual and WoC, Without campaign and consensual. ^+^Denotes cell significantly greater than expected by chance (standardized residual >1.96), ^–^denoted cell significantly less than expected by chance (standardized residual < –1.96). Chi-Square or Fisher’s Exact Test (when there are fewer than 5 counts in any cell) result presented.*

There is also some debate as to whether consent is a psychological or a physical act, in which psychological is based on one’s inner thoughts and physical is based on behavior that represents agreement (Muehlenhard et al., 2016). This debate may be reflected in the current findings because some participants indicated that consent was psychological, for example, not wanting, and others suggested it was a physical act with one participant saying, “I would have said that this is a rape scenario if the “victim” showed indication of wanting to stop.”

In consensual scenarios, the fact that they “both agreed” and “both consented” were most common in suggesting reasons as to why consent was given, and the scenario was therefore consensual. However, many participants still suggested that consent was given in a non-consensual scenario, suggesting the idea that any form of consent is enough (see Supplementary Table S1). Many participants said that “giving in” and “being persuaded” were forms of consent, which can reflect the category of *coercion* in which participants described coercive consent to be both a reason as to why a scenario was consensual as well as why consent was not given.

“Even though there was persistence on one person’s part, the other still gave in, which means that both parties consented before actually having sex.”

Participants often suggested that a non-consensual scenario was consensual because it was wanted and, similarly, suggested that a consensual scenario could have been non-consensual because it was not wanted. Responses may reflect previous research findings that suggest wantedness and consent can be rated as synonymous and also contrasting concepts (Artime and Peterson, 2015) which may suggest a lack of understanding in public perceptions of consent. Participant responses seem to represent *wanting* from the victim rather than the perpetrator in which, if the victim wanted to have sex, then the person has consented to it.

Some participants suggested that *pleasure* influenced their judgment in whether or not a scenario was consensual. Some referred to arousal, pleasure, or “because they enjoyed it” as justification for why a scenario was consensual. Some non-consensual pleasurable scenarios were judged to be consensual with participants suggesting that “bad sex does not mean rape” possibly suggesting that participants believed the common rape myth that women have bad experiences or regret and then “cry rape” (Stewart et al., 1996). Some participants were clear that elements, such as enjoyment, do not represent consent with one participant stating that “pleasure was irrelevant if they didn’t consent.”

“Because even though you didn’t give consent, you enjoyed it.”

It was also found that some non-consensual scenarios were perceived as consensual because the absence of consent was not a “clear message,” which led to the development of the category *communication*. Indeed, there was a significant association between campaign presence and scenario type, in which communication was not mentioned as frequently for non-consensual scenarios for those who had seen the campaign than those who had not. It was also indicated by participants that there needed to be communication of the lack of pleasure or wanting, and participants suggested that the partner cannot “read my mind” but that if intercourse were to continue after it was communicated, it would then depict non-consensual sex. This may also suggest that victim blaming is present in the current findings because participants are suggesting that the victim is at fault for not communicating clearly enough that the person did not consent to sex.

Participants also suggested that being in a *relationship* with a person was often a reason as to why they believed a scenario to be consensual. One participant indicated that “it is just what happens in relationships” and that, therefore, it was not as serious or as likely to be non-consensual compared to if they were not in a relationship. Research has suggested that this rape myth, that consent can be assumed in relationships, is also reflected in barrister opinions in court. A qualitative study found that barristers thought it was not a “terrible offense” if the victim of rape has previously had sex with the perpetrator and suggested that jurors would agree with them (Estrich, 1987; Temkin, 2000). This may also reflect or contribute to the low conviction rate for acquaintance rapes as previously mentioned (Temkin and Krahé, 2008).

Last, although not mentioned as often, *attractiveness* still arose as a category that participants used to suggest why they thought a non-consensual scenario was consensual. Most participants that referred to this in their responses suggested that being attracted to someone or finding their behavior attractive meant that consent could be assumed.

In non-consensual scenarios, the perceived lack of consent present in the scenarios was the most common reason as to why participants thought the scenario was non-consensual. This was often depicted with the phrases “they didn’t agree,” “they refused,” and “they didn’t consent.” In consensual scenarios, consent that was given after *coercion* was used to describe why they perceived the scenario to be non-consensual, especially following the campaign (as evidenced by the significant association between campaign and consent). This suggests the campaign was making participants see coercion in consensual scenarios where they should not have seen it. Reasons such as “scared to say no” and “forced to say yes” were used to represent coercive consent, which some suggested was not consent that was “freely given” and, therefore, did not represent “proper” or “full” consent. However, some participants said that “giving in” or “changing their mind” after coercion was a form of consent and that being uncomfortable due to coercion “does not make it rape.” Coercion, however, was more commonly a reason as to why a situation was non-consensual than why a scenario was consensual (Supplementary Table S1). Some participants showed some confusion and suggested that insistence was not coercive and that the scenario is “not rape unless insistence was coercive,” and others thought the level of coercion was important: “This is difficult without knowing the level of insistence.”

“No true consent can exist under pressure/coercion; the end result makes no difference.”

An interesting finding that arose from the data was the use of second and third person and gender labeling. Despite being asked to imagine themselves in the scenario, second and third person was said more often in non-consensual scenarios than consensual scenarios (see Table 1). This may suggest a form of psychological *distancing* from the scenarios by participants and may suggest that participants may not have wanted to be responsible for a non-consensual scenario and may have wanted to distance themselves and put more responsibility onto the person said to be in the scenario. This may also represent victim blaming because participants may not have wanted to be responsible for the result of the scenario. In addition to this, it was also interesting to see the use of *gender labeling* that appeared in the responses. Both the video and scenarios were gender neutral, but many participants still referred to people in the scenarios as “he” or “she.” It is important to note that the use of “she” was often used to describe the person in the scenario, and “he” was often used to refer to the partner. This could reflect sex-role stereotyping in rape scenarios and demonstrate participants answering with a general sex script in mind that is that females tend to accept or reject a man’s actions and not the reverse (Proite et al., 1993).

### When Was Consent Given?

Table 2 shows the categories for the question regarding when consent was given. Some participants simply stated that consent was not given in a scenario, but for most participants, the point at which consent was given was when the scenario stated either “you agree” or “both people agree.” This supports Beres (2007) findings that the concept of consent is quite well understood as an *agreement* to have sex. There was a difference in the number of mentions between campaign conditions in the agreement category in which there were more mentions of agreement from participants that had not watched the campaign, again possibly suggesting the campaign has produced a rebound effect (Corrigan and Penn, 1999).

**TABLE 2:** Summary content analysis (full analysis presented in Supplementary Table S2) for the question “Please explain at which point in the scenario you think consent was given?”

Category	Description	Mentions				Chi result
		WN	WC	WoN	WoC	
Not Given	This category demonstrates participants suggesting that there was no consent given at any point during the scenario.	209	4	251	7	*X*^2^(2) = 0.36, *p* = 0.761
Coercion	Some students suggested that, in non-consensual scenarios, coercion was the point in which consent was given. This was often indicated by the term “giving in” or “eventually consenting.”	59	7^+^	114	1	*X*^2^(2) = 9.41, *p* = 0.004
Protest	Participants often used a lack of protest by the subject to suggest the point in time when the subject gave consent. Participants suggested that a lack of fight or not displaying disapproval meant that the person had consented.	17	3	22	7	*X*^2^(2) = 0.61, *p* = 0.496
Initiation	Participants suggested that consent was given during the initiation of sex or when sex occurred or began. Some participants suggested that if the subject did not consent to sex then sex would not have occurred and that the occurrence of sex is, therefore, consent.	17^–^	44^+^	45^+^	10^–^	*X*^2^(2) = 33.83, *p* < 0.001
Agreement	Most participants suggested that consent occurred when the two people in the scenario agreed to have sex, came to an agreement, or together said they were OK to have sex, therefore demonstrating that consent occurs when two people agree to take part in sexual activity.	9	391	17	505	*X*^2^(2) = 0.84, *p* = 0.425
Enjoyment	Enjoyment was used by participants to determine a point in time in which consent is given. Participants suggested that when participants enjoyed the experience or began to enjoy themselves, then consent can be assumed.	18	2	29	0	*X*^2^(2) = 3.02, *p* = 0.162
Attraction	Participants have suggested that consent was given when the subject found the other person attractive or found their behavior attractive. There were no participants that stated that consent was given when the person found the partner attractive in consensual scenarios, but in rape scenarios, this was a common point in time for participants to determine when consent was given	8	0	30	0	

*The table contains a description of each category and the number of mentions for each condition in the participants’ responses in their reasons for appraising when consent was given. Condition labels are WN, With campaign and non-consensual; WC, With campaign and consensual; WoN, Without campaign and no-consensual and WoC, Without campaign and consensual. ^+^Denotes cell significantly greater than expected by chance (standardized residual >1.96), ^–^denoted cell significantly less than expected by chance (standardized residual < –1.96). Chi-Square or Fisher’s Exact Test (when there are fewer than 5 counts in any cell) result presented.*

Some participants said that an agreement was given in a non-consensual scenario. This was often when coerced consent was given, which links this category to coercion because many participants suggested that coercive agreement was still an agreement and that consent was given then.

“Consent was given after insistence from the partner.”

The *initiation* of sex or “when sex began” was commonly used to suggest when consent was given. Some participants assumed that because the two individuals in the scenario had sex, then, at some point, consent must have been given but did not specify at what point this may have been. Some participants still said this even when the scenario made the lack of consent quite clear. After seeing the campaign video, participants were more likely to see consent given at the initiation point for consensual scenarios and less likely to see it for non-consensual scenarios, indicating some evidence of the campaign producing insight into when to look for consent.

“Although it says ‘you do not consent’ the person still had sex with them.”

Some participants thought that engaging in sex was consenting (see Supplementary Table S2), which could be a concerning belief to have because the initiation of sex without consent is actually believed by many to be the point at which a scenario becomes non-consensual (Supplementary Table S3) and is also close to the definition of rape (Ministry of Justice Home Office Office for National Statistics, 2013).

Similar to the reasoning as to why a scenario is consensual or non-consensual, categories of *enjoyment* and *attraction* arose from the data. Participants suggested that the point in time at which the victim began to enjoy themselves or began to find the partner or their behavior attractive was the point in time when consent was given. Participants suggested that consent “must have been given” if the person found the experience pleasurable or the behavior of the partner attractive. This highlights the conflation of enjoyment and consent with one participant suggesting that they thought this would also be reflected in the criminal justice system:

“I think a barrister would argue if it was enjoyable it must have been consensual.”

This may suggest that participants think that this opinion is a widely held belief and that others, including those within the legal system who are responsible for defending both victims and perpetrators of rape, also hold the same opinion.

Some participants suggested that “agreeing after persistence” was not “freely” agreeing to take part, and this was not classed as agreeing to take part, and therefore, an agreement was not made, and consent was not given at any point. This reflects the debate present in literature in which some researchers think “any yes” is acceptable as consent (Dripps, 1992) whereas others believe that only “yes means yes” (Beres, 2014). Such a research debate reflects societal opinions. Similar to the first question, *coercion* was falsely made more apparent in consensual scenarios by those who had seen the campaign than those who had not as indicated by the significant chi-square value.

The category *protest* arose from the data demonstrating that consent was given in a scenario at the point when there was a lack of disapproval, for example, “you did not stop it” or “you didn’t say a clear no,” suggesting that, unless one fights against the initiation of sex, you, therefore, agree. This may represent victim blaming by suggesting that it is the victim’s fault for not fighting or not making their rejection clear enough rather than the perpetrators fault for not obtaining consent. Given that the campaign video was designed to combat these kinds of rape myths, it is somewhat surprising to see that it had no significant effect in changing attitudes here.

### When Did the Scenario Become Rape?

Table 3 describes the categories following when the scenario becomes rape. The content analysis indicated that the use of *force* was a point in time when participants suggested a scenario became rape, which reflects previous findings that suggest a forceful scenario is more likely to be perceived as rape (Bachman, 1993; Krahé et al., 2008; Mayers, 2013). The forceful reasoning by participants does not refer to physical force or violence and tends to reflect a verbal force. Force is somewhat described as a more serious case of coercion, which was also one of the most prevalent points in time when a scenario became non-consensual (see Supplementary Table S3).

**TABLE 3:** Summary content analysis (full analysis presented in Supplementary Table S3) for the question “At which point in the scenario do you think It became rape?”

Category	Description	Mentions				Chi result
		WN	WC	WoN	WoC	
Initiation	Participants suggested that the scenario became rape as soon as intercourse occurred in the scenario.	70	3^–^	77	21^+^	X ^2^(2) = 10.40, *p* = 0.001
Coercion	Participants said that it became rape both when coercion began but also when coerced consent was given.	107	10	126	10	X ^2^(2) = 0.12, *p* = 0.817
Force	Force was used to describe scenarios in which they were made to have sex, were forced, the partner insisted, and when force was used. Force does not appear to be described as physical by participants.	122	2	182	0	X ^2^(2) = 2.96, *p* = 0.163
Refusal	Participants suggested that a scenario becomes rape both during and after the rejection of sexual advances.	120	2	129	0	X ^2^(2) = 2.13, *p* = 0.235
Wanting	Few participants described that a scenario became rape when it became an unwanted scenario, often regardless of whether or not sexual advances had been made yet.	8	5	8	4	X ^2^(2) = 0.07, *p* = 1

*The table contains a description of each category and the number of mentions for each condition in the participants’ responses in their answers for when they thought the scenario became rape. Condition labels are WN, With campaign and non-consensual; WC, With campaign and consensual; WoN, Without campaign and non-consensual and WoC, Without campaign and consensual. ^+^Denotes cell significantly greater than expected by chance (standardized residual >1.96), –denoted cell significantly less than expected by chance (standardized residual <–1.96). Chi-Square or Fisher’s Exact Test (when there are fewer than 5 counts in any cell) result presented.*

“When the partner forces sex without consent.”

In terms of *coercion*, participants varied by the point in time at which coercion became non-consensual with some participants suggesting that it became non-consensual “when pressure is put on a person,” whereas others suggested that the scenario became non-consensual when coercive consent was given or when sex was initiated after coercion had occurred: “When sex occurred due to pressure.” “Pressure,” “persistence,” and “persuasion” were often words used to describe coercion that made a scenario non-consensual. This suggests that these participants have different opinions from other participants that suggested that consent was given when coercion occurred, demonstrating the variability in public perceptions (see Table 3).

*Initiation* was also a point in time defined by participants that suggested a scenario became non-consensual as soon as “sex began” or sex was “initiated.” This could reflect knowledge of the meaning of consent because a scenario would be defined as non-consensual only once penetration of the mouth, anus, or vagina had occurred and not before, possibly suggesting that these participants may have knowledge of the definition of rape and, thus, when a scenario may become non-consensual. Those who had not seen the video were significantly more likely to indicate rape occurring at the initiation of consensual scenarios than those who had not seen the video. Similar to coercion, participants suggested that the given scenario became rape “during” *refusal*. Although some referred to the refusal itself, for example, “when you refused to engage in sexual activity,” others suggested that it was when sexual advances continued after refusal and some participants said that it became rape when sex occurred after refusal was given.

“When the partner had intercourse even after the individual refused.”

In line with some participant’s reasons for why they thought the scenario was consensual or not, participants also mentioned *wantedness* and suggested that the scenario became non-consensual when the person did not want to have sex or when intercourse occurred without one or both of the participants wanting to have sex. Wantedness, as previously mentioned, was being conflated with consent and, therefore, highlights the misunderstanding of consent and that there may be little differentiation between the two in public perceptions. This also suggests that participants may have a lack of understanding that someone can consent to an unwanted scenario. Research has suggested there are many reasons for such to occur, such as intimacy, to satisfy partners needs, and to reduce tension (O’Sullivan and Allgeier, 1998).

### When Was Consent Not Given?

Finally, to assess when participants thought consent was not given in scenarios, we ran another content analysis (Table 4). *Agreement* (or lack of) was one of the main reasons as to why consent may have not been given in a scenario and suggested that, when one or both participants do not agree, then consent is not given. Participants who had seen the campaign mentioned agreement significantly less frequently for consensual scenarios than those who had not seen the video. Some participants mentioned that an agreement was not made between the two individuals, and others mentioned that the individual displayed a lack of agreement, such as “refusal” or “rejection.” It could be suggested that these are positive findings because they suggest that people do understand that an agreement is necessary in sexual encounters and that, if such agreement is not achieved, that consent is, therefore, not given, and a scenario represents rape. However, as previously mentioned, consent is not the only factor participants used to determine whether or not consent is given in a scenario (Supplementary Table S1). This category supports Beres (2007) findings that there is a general consensus among the population that consent is an agreement to take part in sexual activity and does suggest that the concept of consent could be quite well understood (Beres et al., 2004).

**TABLE 4:** Summary content analysis (full analysis presented in Supplementary Table S4) for the question “Why do you think consent is not given in this scenario?”

Category	Description	Mentions				Chi square result
		WN	WC	WoN	WoC	
Agreement	Participants mentioned agreement to suggest that consent is not given. Participants suggest that consent is not given when either both or one person doesn’t agree to have sex. Participants suggest this can be shown through agreeing directly to give consent, but not giving consent can be demonstrated in other ways such as refusing or rejecting sex.	136	2	170	14	*X*^2^(2) = 6.34, *p* = 0.017
Choice	The lack of freedom and choice to be able to give consent was a category that arose at to why consent would not be given. Participants often mentioned that, although consent was given in some scenarios, this was not out of choice, which therefore, led to participants saying that consent given without freedom of choice was not “real” consent.	10	1	17	2	*X*^2^(2) = 0.02, *p* = 1
Wanting	Participants often used wanting and consenting interchangeably in previous questions and, therefore, not wanting to have sex was often one of the reasons as to why consent was not given. In scenarios that were consensual but unwanted, participants often suggested that consent was not “proper” consent.	16	1	14	5	*X*^2^(2) = 2.70, *p* = 0.182
Coercion	Coercive methods were referred to when participants suggested why they thought consent was not given in a scenario. Strategies, such as persuasion, pressure, and persistence came under the category of coercion. Some participants mentioned that “true” consent cannot exist under pressure or coercion.	33	4	59	6	*X*^2^(2) = 0.07, *p* = 1
Fear	Participants suggested that consent was not given in some scenarios because the subject was scared of the other person or the consequences of rejection. They often referred to the fear making the consent not “proper” consent.	0	6	0	14	

*The table contains a description of each category and the number of mentions for each condition in the participants’ responses in their reasons for thinking consent is given or not given. Condition labels are WN, With campaign and non-consensual; WC, With campaign and consensual; WoN, Without campaign and non-consensual and WoC, Without campaign and consensual. Chi-Square or Fisher’s Exact Test (when there are fewer than 5 counts in any cell) result presented.*

“Both you and your partner agree to do so.”
“I don’t think consent is given freely; therefore, it is not properly given.”

A lack of freedom to give consent was mentioned by participants and suggested that, although consent was given in a scenario, the person who gave the consent did not give consent with the *freedom* to choose meaning that participants believed it to be non-consensual, which suggests participants may be aware that, by definition, consent needs to be given with free choice (Home Office, 2003). Participants also said that many scenarios were non-consensual because they were *unwanted*. This, again, highlights the possible confusion between wanting and consent.

“It wasn’t what you wanted to happen. You didn’t want sex.”

*Coercive* methods were also referred to, suggesting that the use of coercive strategies, such as pressure and persuasion could suggest why consent was not given, however, it should be noted that coercive methods have previously been mentioned as a reason as to why a scenario was consensual (Table 3), which suggests a difference in perceptions across participants. Participants who used coercion to suggest why consent was not given in a scenario suggested that “true” consent cannot be given under pressure, which is in line with some researchers’ definitions of consent (Humphreys, 2004).

“Consent is given when the person agrees to have sex, but it’s not free consent in the same way someone who initially wanted to have sex gives consent.”

Some suggested that consent was not given in a scenario because of the presence of *fear* in the individual in the scenario; this was often related to the consequences of the sexual act and being “scared of the repercussions of saying no.” “How the partner may react” was also referred to by participants. Participants mentioned that simply being fearful of the partner themselves may suggest a reason as to why consent was not given. Additionally, fear was only mentioned in coercive consensual scenarios, suggesting that the consent given in the scenarios was not “proper” consent. This may be because there were more “clear cut” reasons as to why consent was not given in scenarios that were not coerced or non-consensual, such as lack of agreement.

“Although they agreed, they agreed because they were scared and were trying to protect themselves from danger.”

## Conclusion

The quantitative findings have shown that the campaign video was not effective given that participants who had seen the campaign gave similar responses to those who had not. This supports previous findings that media campaigns may not be effective in changing public attitudes (Ogunfowokan and Fajemilehin, 2012; DeGue et al., 2014; Hills et al., 2020) and that attitudinal changes in society can be difficult to achieve (Brecklin and Forde, 2001). The qualitative analysis seemed to offer a potential explanation for this. Consent was actually mentioned more often by those who had *not* seen the campaign, suggesting some sort of rebound effect. It might reflect that participants acted contrary to the instructions in the video. Of course, we were only considering participant identification of rape rather than actual behaviors. Further, the viewing of the video was immediately before reading the sexual scenarios. Nevertheless, we would have expected more convincing findings regarding the effectiveness of the campaign if it was processed deeply by our participants.

A possible reason as to why the campaign was not effective may be due to the lack of realistic elements in the video. The campaign showed stick men to represent humans as well as tea to represent consent. Because both of these elements are very different from how real-life rapes occur, it is possible that it could have been hard for participants to relate the video to the scenarios. This, however, could have increased the external validity of the video campaign because it is unlikely that media campaigns are able to demonstrate exactly what will happen in a real-life rape, which means, for such campaigns to be effective, the resulting attitudinal change needs to be applicable and generalizable to other non-consensual examples (Camilleri et al., 2015). A second reason is that there is no reason for our participants to engage with the video during its presentation (Fabiano et al., 2003; Binet and Field, 2009). They were simply instructed to watch it (similar to if it were on TV or given during a university induction event). If the participants were asked to discuss the video with others, potentially it would have led to more deep coding and analysis and more attitudinal change. Further, the scenarios depicted, although representative of the most common type of sexual assault, are not consistent with stereotypical rape (Stirling et al., 2020). When the message is further from one’s internal script, it is harder to process the message (Neuschatz et al., 2002) meaning that, without elaboration, the “Tea and Consent” video may not be remembered. However, because little is known about what makes a campaign effective in changing attitudes and behaviors long term (Lonsway, 1996) we can only speculate that these elements may have contributed to the lack of effectiveness of this media campaign.

Potentially a more disturbing effect of the video was that it appeared to cause participants to discuss coercion in consensual sexual scenarios. This indicates that, although the video did not encourage participants to use consent in their understanding, it made them see coercion where it was not present. This may be a demand characteristic (participants felt they should be looking for more coercive behaviors in the scenarios following seeing the video), but if so, one would expect to see coercion discussed more in the non-consensual scenarios as well. This was not the case. Furthermore, seeing the video made people less likely to suggest the initiation of sex in non-consensual scenarios was when rape occurred than not seeing the video. This evaluation point highlights a clear need to properly evaluate how people interpret and use campaign videos.

More broadly, the content analysis of the free-text responses gave an insight into public perceptions around rape and consent. Although there is some coherent understanding in some areas, such as the need for agreement, there is still some confusion in many aspects, particularly the conflation of wanting and consent as well as high variability in opinions regarding coercive consent and the concept of “real” consent. Many participants referred to the scenarios as highlighting not “proper,” “full,” or “real” consent. This may suggest that participants think that consent is not a simple dichotomy as implied by the law. Participants see consent along a spectrum depending on various factors in the scenario, such as fear, coercion, wanting, or choice.

The conflation of consent and wantedness has arisen throughout the content analysis and may suggest a possible avenue to develop training for jurors in rape cases to help them to better discriminate between wanting and consenting in sexual scenarios to increase understanding and, thus, conviction. This will help to ensure that all sexual assaults that occur without consent are treated as such in court. Future research may wish to investigate effective methods to inform participants and, therefore, potential jurors about the differences between wanting and consenting.

Future research may wish to look into alternative methods to change public attitudes and should focus on a more rounded approach to target more than just rape awareness and to target victim empathy, rape myths, and demonstrate the consequences of rape (Schewe, 2002). Schewe (2002) has suggested these elements are necessary for effective rape prevention programs. This may lead to more empathetic perceptions toward victims of rape as well as being better informed of the consequences and the prevalence of rape. This may lead to a change in both public attitudes toward rape and the occurrence of rape.

Throughout our analysis, we have ignored several individual difference factors that may influence understanding of consent and rape. This was deliberate because we did not collect demographic data. Although this was intended to increase the anonymity and honesty for participants, it means we are unable to assess the representativeness of our sample. Further, we were unable to assess gender differences in response to the campaign and possible gender differences in perceptions of the scenarios. Gender differences are generally present in the perceptions of sexual scenarios and sexual abuse (Schutte and Hosch, 1997; Hockett et al., 2016; James, 2018), however, in the scenarios we used, gender differences have typically not been found (Hills et al., 2020). Nevertheless, this limitation should be acknowledged.

Question order was found to be effective in changing the judgments of rape, and the results show that highlighting consent before asking participants to make a judgment on rape significantly increased the number of rape judgments. The implications of this result are twofold. In the first, it highlights that participants do not fully realize that rape is defined as sex without consent. Second, when participants are explicitly made aware of the lack of consent, they understand that a scenario depicts rape, indicating unconscious knowledge of the definition of rape. The results demonstrate that the problem may not lie within the knowledge of consent because results show that, once consent has been highlighted, participants were able to better identify it. Although the qualitative content analysis does suggest some misunderstanding, it may be that the findings have provided implications for jurors in the decision-making process in court. This could be difficult to implement due to the strict and structured judicial system and jurors’ decision making process (Pennington and Hastie, 1986), however, it highlights the need to inform jurors that consent is all that matters in the judgment of rape. The primacy of this knowledge can impact on the accuracy of rape judgments. This could, in turn, encourage reporting of rapes because many victims of rape do not report due to the fear of not being believed (Sable et al., 2006); therefore, if victims feel like they are more likely to believed and convict their perpetrator, it may lead to progress in increasing reporting and conviction.

## Data Availability Statement

All datasets generated for this study are included in the article/Supplementary Material.

## Ethics Statement

The studies involving human participants were reviewed and approved by the Bournemouth University Research Ethics Committee. The patients/participants provided their written informed consent to participate in this study.

## Author Contributions

ER: design and implementation of the study, data analysis, and drafting of the work. PH: supervision, guidance on study design, corrections on drafts, and some data analysis. All authors contributed to the article and approved the submitted version.

## Conflict of Interest

The authors declare that the research was conducted in the absence of any commercial or financial relationships that could be construed as a potential conflict of interest.

## Footnotes

1 https://www.youtube.com/watch?v~=~oQbei5JGiT8
