# Supplementary material for: The Effect of Passively Viewing a Consent Campaign Video on Attitudes Toward Rape
Source: Front Psychol. 2020 Jul 28;11:1741. doi: 10.3389/fpsyg.2020.01741 (PMC7399096; doi:10.3389/fpsyg.2020.01741)
Supplement: Supplementary file 1 [file Table_1.DOCX]

Table S1.

*Content analysis table for the question ‘Please explain why you think this scenario is rape or not rape’. Table includes content categories, the number of times the category was mentioned as being used to make judgments about when consent was given in consensual scenarios or when consent was not given in non-consensual scenarios (Positive mentions) and the number of times the category was used to make judgments about when consent was given in a non-consensual scenario or when consent was not given in a consensual scenario (Negative mentions), an explanation of how the category was used and example quotes.*

| Category | Positive Mentions | Negative Mentions | Descriptions | Example of Transcripts |
| --- | --- | --- | --- | --- |
| Consent | 1692 | 259 | Consent was used by most participants to describe why they thought a given scenario was rape or not rape. This seemed to be the key factor in determining whether or not a scenario was rape. Some participants stated that absence of informed consent meant a scenario was rape, whilst others said that “giving in” and “changing their mind” after coercion was still consent. Consent and wanting also appeared to be used interchangeably as if consent and wanting are the same thing. | “It isn’t rape because both parties consent” (Consensual scenario)  “Saying that you ‘gave in’ is almost as though they have consented in a way. it does feel as though they eventually said yeah” (Rape scenario) |
| Wanting | 99 | 31 | Wanting was used as reasoning for both consensual and non-consensual scenarios in which consensual were consensual because they were wanted and rape scenarios were rape because they were unwanted. It appeared that some participants determined whether or not a scenario was rape or not based on whether or not it was wanted, whilst others used consent and wanting synonymously. | “It is not rape, because the person does want to have sex” (Rape scenario)  “Partner has insisted on having sex even though you didn’t want to” (Rape scenario)  “As they both wanted to have sex” (Consensual scenario) |
| Pleasure | 220 | 116 | Pleasure was used by participants in order to perceive a scenario as consensual or not. Some participants stated that the enjoyment experienced in a scenario is irrelevant if a scenario is non-consensual, whilst others based their decisions on pleasure with some suggesting that enjoying the experience was the factor that lead them to perceive the scenario as consensual. | “Both agree to sex and enjoy it” (Consensual scenario)  “not rape - you engage in sexual activity and enjoy it” (Rape scenario) |
| Communication | 101 | 0 | Communication was used by participants to highlight the idea that consent or pleasure should be vocalised in order to make a decision. Communication was used by participants to suggest that an unwanted or unpleasable scenario should be vocalised or discussed since the partner will otherwise not be aware. This was despite consent. Participants suggested that if the unwantedness or pleasure was vocalised and intercourse continued, that they may then consider it to be rape. | “Even though you did not want to have sex to begin with, the other person cannot know this unless you tell them and you agreed to have sex with them so consent is given and it therefore, is not rape.” (Consensual scenario)  “If you made your feelings aware to the other person and they carried on I do believe this would be rape. If you're uncomfortable with someone's feelings, you should say something.” (Consensual scenario) |
| Coercion | 472 | 183 | Coercion was used by participants for reason as to both why a scenario may be consensual or non-consensual. Those who used coercion for reason as to why it was non-consensual sometimes described the consent as not “real” or ‘proper’ which may suggest why. Others considered any agreement to be consent, including those agreements and consents that were made under or as a result of coercion. | “Saying that you ‘gave in’ is almost as though they have consented in a way” (Rape scenario)  “It could be classed as rape as the partner persisted with trying to initiate sex” (Consensual scenario)  “The person persisted and persuaded them to have sex despite being rejected” (Rape scenario) |
| Distancing | 1043 | N/A | Participants often used third person tenses in their responses. Participants appeared to distance themselves to scenarios and this occurred more so in rape scenarios than consensual scenarios. Participants often used “they”, “you”, “the person” or “the individual” to describe the person in the scenario that they were asked to imagine themselves being and answer accordingly. | “Even though they didn’t want to they still agreed.”  “It can be considered rape as the person did not consent to have sex.”  “As you consented to it.” |
| Gender Labelling | 62 | N/A | Gender labelling was found to appear in some participant responses. Participants often used gender labels in their responses using “he” and “she” most commonly. Participants who used gender labels very rarely said “he/she” and often labelled the victim of the consensual or rape scenario as the ‘she’ and the partner in the scenario as “he”. | “She refused intercourse and he continued”  “she said no - and he knew why” |
| In a relationship | 39 | 0 | Being in a relationship was an element of the scenarios that some participants suggested was a reason as to why a non-consensual scenario was not rape. They often suggested that these things happen in relationships and therefore need communication, rather than that these scenarios are rape. | “Because he's my partner it's what happens.” (Rape scenario)  “Because he is my partner, and probably it was just an incident which happens in relationships”(Rape scenario) |
| Attractiveness | 2 | 17 | Attractiveness was mainly used as reasoning in rape scenarios as to why it is not rape, rather than why a consensual scenario is consensual. Participants have suggested that being attracted to someone and finding their behaviour attractive and appealing may lead them to reason that a rape scenario may be consensual, since you are attracted to the person | “Consent initially not present but appears to have then been given because they find partners behaviour 'appealing and attractive'”  “They found their behaviour attractive and appealing” |

Table S2.

Content analysis table for the question ‘At which point in the scenario, if any, do you think describes when consent is given?’. Table includes content categories, the number of times the category was mentioned as being used to make judgments about when consent was given in consensual scenarios or when consent was not given in non-consensual scenarios (Positive mentions) and the number of times the category was used to make judgments about when consent was given in a non-consensual scenario or when consent was not given in a consensual scenario (Negative mentions), an explanation of how the category was used and example quotes

| Category | Positive Mentions | Negative Mentions | Descriptions | Example of Transcripts |
| --- | --- | --- | --- | --- |
| Not given | 460 | 11 | This category simply demonstrates those participants that thought consent was not given at all in the scenario. | “Consent was not given.” |
| Coercion | 14 | 165 | A Positive mention of coercion means a participant suggested that in a rape scenario, coercion meant consent was not given. A negative mention suggests that in a rape scenario consent was given at some point during or after coercion. | “agreement following partner's insistence.” (Rape scenario)  “giving in to persistence.” (Consensual scenario) |
| Protest | 10 | 30 | The category protest demonstrates that participants often thought a scenario was not rape when there was a lack of disapproval shown by the subject in the scenario. Positive mentions demonstrate a lack of protest in consensual scenarios. | “You didn't refuse after partner insists” (Rape scenario)  “you could have carried on saying no” (Rape scenario) |
| Initiation | 54 | 62 | Participants suggested that consent was given when sex was initiated. Participants have said this in both consensual and rape scenarios. | “The initiation and continuance of mutually agreed sexual contact”  “At the initiation stage” |
| Agreement | 898 | 24 | Most participants said that consent occurred during agreement in a consensual scenario, however some participants suggested that an agreement occurred in a rape scenario. | “Both partners agreed to do so.” (Consensual scenario)  “you agreed to it and enjoyed the experience” (Rape scenario) |
| Enjoyment | 2 | 47 | Enjoyment was used by participants as a point in time in which consent was given in rape scenarios. Participants suggested that when the subject started to enjoy themselves, consent was then given. It could be assumed that participants meant non-verbal consent. | “Probs after sex has started and I've started enjoying myself.” (Rape scenario)  “no point but it is implied by the enjoyment.” (Rape scenario) |
| Attraction | 0 | 38 | In rape scenarios, participants suggested that consent was given when subjects found their partner attractive. | “when you find your partners behaviour appealing and attractive in the situation.” (Rape scenario) |

Table S3.

Content analysis table for the question ‘At which point do you think this scenario became rape?’. Table includes content categories, the number of times the category was mentioned as being used to make judgments about when consent was given in consensual scenarios or when consent was not given in non-consensual scenarios (Positive mentions) and the number of times the category was used to make judgments about when consent was given in a non-consensual scenario or when consent was not given in a consensual scenario (Negative mentions), an explanation of how the category was used and example quotes.

| Category | Positive Mentions | Negative Mentions | Descriptions | Example of Transcripts |
| --- | --- | --- | --- | --- |
| Initiation | 171 | N/A | Participants suggested that the scenario became rape when sex began or when sex was initiated. As soon as intercourse began to occur without consent, participants suggested that this is when the scenario became rape. Some participants referred to the fact that the person carried on anyway, others highlighted that it was without consent whilst others just stated that initiation of intercourse is when the scenario became rape. | “When sex begins.”  “When sex without consent occurred.” |
| Coercion | 253 | N/A | Participants suggested that the scenario became rape both at the point when coercion began but also when coerced consent was given. Persistence, pressure and persuasion were often mentioned as describing words demonstrating coercion. Participants thought that. | “When they begin to persist.”  “when sex occurred without true consent.”  “the person uses pressure = coercion - whether physical, verbal or emotional.....” |
| Force | 306 | N/A | Force was used by participants to describe the point in time when the scenario became rape. Participants often suggested that sex being forced was an indication of rape but also when consent was forced. Participants also suggested that when the choice to have sex was taken away from the subject, it then became rape as they were then forced. | “The person forced you to have sex.”  “when force was used to get the other person to engage in the act.” |
| Refusal | 251 | N/A | Many participants suggested that the scenario became rape when sex was rejected or refused rather than at the time when sex commenced. However, others suggested that it was any sexual activity that happened after refusal or rejection that made the scenario rape. | “when the partner continued even after the refusal.”  “when you refused to engage in sexual activity.” |
| Wanting | 25 | N/A | A few participants described that a scenario became rape at the point where it became unwanted. Sometimes participants simply referred to not wanting and did not refer to whether or not any sexual advances had been made. | “when they had sex with one person not wanting it.”  “When the person doesn't want to have sex.” |

Table S4.

Content analysis table for the question ‘Why do you think consent is not given in this scenario?’. Table includes content categories, the number of times the category was mentioned as being used to make judgments about when consent was given in consensual scenarios or when consent was not given in non-consensual scenarios (Positive mentions) and the number of times the category was used to make judgments about when consent was given in a non-consensual scenario or when consent was not given in a consensual scenario (Negative mentions), an explanation of how the category was used and example quotes.

| Category | Positive Mentions | Negative Mentions | Descriptions | Example of Transcripts |
| --- | --- | --- | --- | --- |
| Agreement | 322 | N/A | Participants mentioned agreement to suggest that consent is not given. Participants suggest that consent is not given when either both or one person doesn’t agree to have sex. Participants suggest this can be shown through agreeing directly to give consent but not giving consent can be demonstrated in other ways such as refusing or rejecting sex. | “No agreement is made.”  “Rejected when consent was requested.” |
| Choice | 30 | N/A | The lack of freedom and choice to be able to give consent was a category that arose as to why consent would not be given. Participants often mentioned that whilst consent was given in some scenarios, this was not out of choice which therefore led to participants saying that consent given without freedom of choice was not ‘real’ consent. | “’Giving in’ makes it seem like the person was not given a choice.”  “You are not free to choose what you want.”  “You gave in due to pressure not choice.” |
| Wanting | 36 | N/A | Participants often used wanting and consenting interchangeably in previous questions and therefore not wanting to have sex was often one of the reasoning as to why consent was not given. In scenarios that were consensual but unwanted, participants often suggested that consent was not ‘proper’ consent. | “It wasn’t what you wanted to happen. You didn’t want sex”  “Proper consent is not given, sex is not wanted” |
| Coercion | 102 | N/A | Coercive methods were referred to when participants suggested why they thought consent was not given in a scenario. Strategies such as persuasion, pressure and persistence came under the category or coercion. Some participants mentioned that ‘true’ consent cannot exist under pressure or coercion. | “There is the coercion and the persistence to have sex with their partner.”  “There can be no true consent under pressure or coercion.” |
| Fear | 20 | N/A | Participants suggested that consent was not given in some scenarios because the subject was scared of the other person or the consequences of rejection. They often referred to the fear making the consent not “proper” consent. | “Because although they agree it is done out of fear.”  “Because even though they agreed to have sex, they were doing so out of fear of the other person.” |
